# Novel Feature Generation for Classification of Motor Activity from Functional Near-Infrared Spectroscopy Signals Using Machine Learning

**DOI:** 10.3390/diagnostics14101008

**Published:** 2024-05-13

**Authors:** V. Akila, J. Anita Christaline, A. Shirly Edward

**Affiliations:** Department of ECE, SRM Institute of Science and Technology, Vadapalani, Chennai 600026, India; anitaj@srmist.edu.in (J.A.C.); edwards@srmist.edu.in (A.S.E.)

**Keywords:** independent component analysis (ICA), functional near-infrared spectroscopy (fNIRS), machine learning, motor activity, feature extraction

## Abstract

Recent research in the field of cognitive motor action decoding focuses on data acquired from Functional Near-Infrared Spectroscopy (fNIRS) and its analysis. This research aims to classify two different motor activities, namely, mental drawing (MD) and spatial navigation (SN), using fNIRS data from non-motor baseline data and other motor activities. Accurate activity detection in non-stationary signals like fNIRS is challenging and requires complex feature descriptors. As a novel framework, a new feature generation by fusion of wavelet feature, Hilbert, symlet, and Hjorth parameters is proposed for improving the accuracy of the classification. This new fused feature has statistical descriptor elements, time-localization in the frequency domain, edge feature, texture features, and phase information to detect and locate the activity accurately. Three types of independent component analysis, including FastICA, Picard, and Infomax were implemented for preprocessing which removes noises and motion artifacts. Two independent binary classifiers are designed to handle the complexity of classification in which one is responsible for mental drawing (MD) detection and the other one is spatial navigation (SN). Four different types of algorithms including nearest neighbors (KNN), Linear Discriminant Analysis (LDA), light gradient-boosting machine (LGBM), and Extreme Gradient Boosting (XGBOOST) were implemented. It has been identified that the LGBM classifier gives high accuracies—98% for mental drawing and 97% for spatial navigation. Comparison with existing research proves that the proposed method gives the highest classification accuracies. Statistical validation of the proposed new feature generation by the Kruskal–Wallis H-test and Mann–Whitney U non-parametric test proves the reliability of the proposed mechanism.

## 1. Introduction

Functional near-infrared spectroscopy (fNIRS) is a non-invasive and less expensive alternative method to study brain activities compared to functional magnetic resonance imaging (fMRI) and positron emission tomography (PET). It has gained popularity in the recent past. Previous research has mostly focused on enhancing fNIRS sensitivity and specificity for therapeutic purposes. Scholkmann et al. [1] provided a thorough review and explanation of the advantages of fNIRS imaging methodology and the instruments. They also discussed the restrictions of fNIRS. Despite a few limitations, fNIRS seems to be promising in acquiring brain signals during brain activities [2]. To fully realize the promise of fNIRS and to overcome the obstacles preventing its broad application in neuroscience, fNIRS signal processing techniques need more attention. An insufficient grasp of the underlying physiology has resulted from previous research’s frequent use of signal processing and data analysis approaches that do not completely account for the complicated relationships between physiological factors. To solve this difficulty, more advanced techniques like machine learning that combine a more extensive knowledge of the underlying physiology and the relationships between different physiological factors are required. The accuracy and reliability of fNIRS data analysis may be increased, and information about how the brain functions in both healthy and pathological situations can be achieved only by creating better data-processing techniques [3]. 

This research work intends to find the best fNIRS signal processing methods that use machine learning to improve fNIRS-based brain studies, specifically motor activity.

## 2. Literature Review 

Machine learning applications in functional near-infrared spectroscopy (fNIRS) have been the topic of multiple research efforts in recent years. The main focus is to classify the fNIRS signals acquired during human–computer interactions to identify the different brain activities. Another area of growing research in this field is using deep learning algorithms to discover significant patterns in fNIRS data. A methodology called fNIRS-QC was implemented by Gabrieli et al. [4] to crowdsource the creation of a quality control fNIRS dataset. The authors used a dataset of 1340 fNIRS signals from 67 individuals and studied the detection rate for 548 segments. A machine learning model was created to assist researchers in estimating the quality of a single signal segment [5]. They developed a machine learning model for finding a biomarker of human pain using fNIRS for the non-invasive evaluation of human pain. They infer that machine learning technologies have important implications for the development of innovative pain management solutions. Researchers can acquire insights into the usefulness of different approaches for pain treatment by assessing the performance of these algorithms. This model has the potential to introduce advancements in pain treatment innovation and enhance the life of people dealing with chronic pain. Other research about fNIRS-based motor cognition include a comprehensive and flexible vision framework [6], categorizing motor activity execution [7], multi-level Alzheimer’s disease classification conducted using deep learning [8], and fNIRS-based brain–computer interfaces were used for comparison of various classification methods in a two-class system [9]. All these approaches were used in the context of classifying data in the field of fNIRS. A machine learning-based method named neural data augmentation was presented as a solution for increasing the spatial resolution of fNIRS and making it more widely used as a supplementary technique to fMRI [10]. Deep neural networks and other machine learning models have also been used to map fNIRS to functional magnetic resonance imaging (fMRI) [11], but this method only achieves an R2 value of a maximum of 0.444 which means it is not able to capture the variance in the independent variable. 

Deep learning techniques are utilized for task-based classification of fNIRS signals [12] linked to hand gestures like rock, paper, and scissors. The application of convolutional neural network (CNN) in the processing of fNIRS data during rock, paper, and scissors hand gestures has been investigated. The time series classification (TSC) algorithm with the CNN model is able to achieve 97% accuracy only. There is a need for improvement in accuracy. Another study by Wickramaratne and Mahmud [13] used fNIRS data to construct a deep learning-based classification system that can properly categorize ternary activities including basic math, motor function, and the rest state. For fNIRS data acquisition, they applied a deep convolutional neural network (DCNN) and a feature extraction method known as the Gramian Angular Summation Field (GASF) [13]. The system can only achieve 87.14% average classification accuracy. There is a need for improvement in accuracy. Furthermore, the efficiency of diverse machine learning algorithms including decision trees, support vector machines, and artificial neural networks was examined to identify mental workload levels. This evaluation used fNIRS data obtained from individuals performing human–computer interaction tasks with varying degrees of mental work were studied by Nagels et al. [14]. Joint oxygenated hemoglobin (HbO) signal and the deoxygenated hemoglobin (HbR) signal the analysis of all five encoding trials given an average accuracy of 66.67%. When used on half of the participants, accuracy improved to 83.33% using either the HbO signal or the HbR signal. When both signals are involved, the model is not able to achieve good performance.

Independent component analysis (ICA) is a pre-processing technique used for separating unwanted signals from original signals, feature extraction, and noise removal using artifacts. Zhang et al. [15] used principal component spatial filtering to build a pre-processing approach for functional near-infrared spectroscopy (fNIRS) data principal component spatial filter (PCSF). To reduce noise and improve signal quality, the approach separates the global and local components of the fNIRS signal. The findings indicate that PCSF successfully minimizes noise from physiological and motion aberrations and that it may be valuable in increasing the accuracy of fNIRS-based brain imaging. Different types of ICA methods such as Picard and extended infomax are applied to EEG signals for effective signal separation and the removal of muscle components from desired EEG signals [16]. The ICA method has proved its efficiency in decreasing global noise in fNIRS data [17]. A novel user-friendly Matlab Toolbox called NIRS-ICA has been used by researchers for effective signal separation from source [18]. 

Multimodal deep learning analysis using fNIRS and electroencephalogram (EEG) has been employed by Ortega, P., and Faisal [19] to study the bimodal force exerted by the fingers to hold things, such as motor coordination and control. Deep learning has also been implemented for refining workload classification in adaptive human interfaces [20,21], only automatic identification of cognitive fatigue schedules [22] with a maximum classification accuracy of around 70.91 ± 13.67% was achieved, and classification based on single and dual walk tasks [23] in older adults. Machine learning analysis has also been carried out by many researchers for studying unique brain patterns in connection with migraines during mental tasks [24], the age-related hearing problem tinnitus [25], and the classification of healthy children and Attention Deficit Hyperactivity Disorder (ADHD) in children [26]. Time–space analysis of task-based classification was conducted by Trakoolwilaiwan et al. [27] based on multivariate time series analysis for three tasks, namely, right, left, and in resting state using CNN. Huve et al. [28] implemented classification based on support vector machine (SVM), long short-term memory (LSTM), and LDA for tasks such as counting, arithmetic, and solving puzzles. Classification of the fNIRS signal based on three mental tasks such as rest state, subtraction, and generation of words using LSTM and CNN was studied by Asgher [29,30]; their results were improved and compared to classical machine learning methods [31]. Recurrent CNN showed classification of both time and spatial signals with good accuracy of EEG signals [32]. 

Feature extraction and classification of bimodal fNIRS data representation by selecting cognitive and motor disability patients were analyzed by Hong et al. [33] for specific tasks related to word formation and arithmetic tasks and classification using vector phase analysis. Graph convolutional network (GCN) has been used for channel selection and feature extraction for four-class classification [34]. Within and between class distance factorization calculated is classified using a random forest classifier by Zhong et al. [35]. The Stroop task was classified using classical machine learning algorithms such as support vector machine (SVM), artificial neural network (ANN), k-nearest neighborhood (KNN), and decision forest by Cuong [36]. 

Even though there are machine learning- and deep learning-based works for the classification of mental activity, the accuracy achieved by them is not up to remarks. The existing methods are not able to achieve high accuracy since the classification of non-stationary signals such as fNIRS requires complex feature descriptors. So, this research intends to: 

Generate a new feature set using wavelet, Hilbert, and Hjorth parameters to increase the accuracy. Accurate activity detection in non-stationary signals like fNIRS is challenging and requires complex feature descriptors. As a novel framework, a new feature generation by fusion of wavelet feature, Hilbert, symlet, and Hjorth parameters are proposed for improving the accuracy of the classification. This new fused feature has statistical descriptor elements, time-localization in the frequency domain, edge feature, texture features, and phase information to detect and locate the activity accurately. 

Uses independent components analysis to remove the artifacts. 

New feature-based classification using a machine learning technique is proposed to be used to distinguish between two different motor tasks, namely, mental drawing (MD) and spatial navigation (SN) from non-motor baseline data and other motor activity.

Two independent binary classifiers are designed to handle the complexity of classification in which one is responsible for mental drawing (MD) detection and the other one is spatial navigation (SN). 

## 3. Materials and Methods

**Figure 1:**
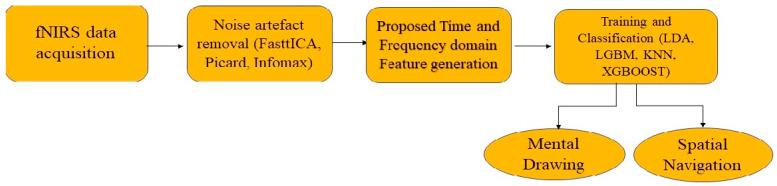
Block diagram of proposed method which uses independent component analysis for artifact removal and newly generated feature, classical time, and frequency domain feature for classification.

This research aims to address these issues of improving classification accuracy by using new feature generation. Three different methods of independent component analysis (ICA), namely FastICA, Picard, and Infomax for the removal of noise artifacts are used followed by four different machine learning classifiers. These classifiers include LDA, KNN, LGBM, and XGBOOST. The noise handling new feature generation using wavelet, Hilbert, and Hjorth parameters are implemented in this research to identify the best accuracy in detecting motor tasks such as spatial navigation (SN) and mental drawing (MD). Wavelets are used to handle motion artifact correction [37], phase information of fNIRS signals extracted using Hilbert transform to detect the activity [38]. Also, the classical statistical time domain and frequency domain features are also used. The following figure shows the overall proposed methodology. The acquired fNIRS signal is initially pre-processed with different noise-removal techniques as in Figure 1.

The study specifically used optode configurations for acquiring fNIRS data without physiologically separated channels, which would have negatively impacted the signal quality and added physiological noise to the fNIRS data. Hence, noise removal is implemented.

### 3.1. fNIRS Motor Imagery/Movement Dataset

The data used in this investigation were taken from a prior study conducted by Nagels-Coune et al. [14]. The study comprised 18 healthy volunteers with normal hearing and 8 female participants, with an average age of 26.00 ± 8.19 years (mean ± SD). Nagels-Coune et al. [14] obtained permission from all participants before the experiment, and this study received approval from the local ethics committee adhering to the principles of the Helsinki Declaration. Participants in this study were given a full informed consent process before being presented with two mental imagery activities. Participants were asked to visualize sketching basic shapes with their right hand in the first activity for the mental drawing (MD) (or non-dominant hand for left-handed participants) activity. Spatial navigation (SN), the second test, asked participants to envision going around a familiar area and perceiving the visuals in each room. Before the trial, participants were asked 45 binary questions, with 6 of them (3 “yes” responses and 3 “no” responses) selected for the primary fNIRS experiment.

### 3.2. Methodology

There are three main phases involved in this research. First is data collection; second is data pre-processing (artifact removal); third is new feature generation and classical feature generation and finally classification process to classify the signal into mental drawing or spatial navigation. Each of these phases is explained in this section.

#### 3.2.1. Data Collection

The dataset was obtained by written consent from Nagels-Coune [14]; this dataset consists of 18 healthy participants (age: 26.00 ± 8.19 years (mean ± SD)). The two mental imagery activities considered in this research are as follows:

First task: visualization of sketching basic shapes with their right hand in the first activity—mental drawing (MD).

Second task: participants go around a familiar area and perceive the visuals in each room—spatial navigation (SN).

During the experiment, hemodynamic data were captured using a continuous-wave fNIRS system (NIRScout-816, NIRx Medizintechnik GmbH, Berlin) and NIRStar software (version 12.0, NIRx Medizintechnik GmbH). The sampling rate was 12.5 Hz [14]. The system included three light-emitting LED source optodes with wavelengths of 760 and 850 nm along with six detector optodes. These nine optodes were positioned in accordance with the worldwide 10–20 EEG method, with three source optodes located on FC3, C3, and CP3 positions, and six detector optodes on FC5, C5, CP5, FC1, C1, and CP1. Each source and detector optode pair constituted a separate channel for a total of 18 channels. Nevertheless, four channels (FC3-CP1, FC3-CP5, CP3-FC1, and CP3-FC5) were omitted from the investigation because the inter-optode distance exceeded 60 mm. The owners of the dataset [14] state that 4 out of 18 channels are neglected based on distance.

Their dataset has been used by many researchers and hence we assume that the dataset is valid without any lack of control when recording is under natural conditions, has proper signal separation, satisfies the diverse factors such as age, health conditions, and brain morphology that could affect the applicability of the findings to different scenarios and other variabilities.

The authors of the dataset [14] state that the participants were allowed to relax in between the recordings by changing body position or drinking water. The fNIRS cap was removed after 10 runs to increase the pleasantness of the recording of the MD and SN tasks. Also, they were asked to rate their level of comfort on a point scale of 10.

**Figure 2:**
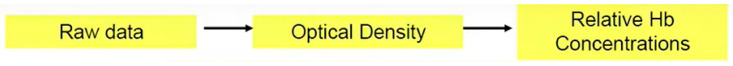
Conversion of raw fNIRS signal to Hb concentrations: raw data are converted into optical density then converted into hemoglobin.

The fNIRS data acquired are converted into optical density values according to the modified Beer–Lambert law. This sequence of operation is shown in Figure 2 and the corresponding formula is as shown in Equation (1).

(1) $$OD\left(t,\lambda \right)=-{log}_{10}\left(\frac{I\left(t,\lambda \right)}{I_{o}\left(t,\lambda \right)}\right)=\sum_{i}\varepsilon_{i}\left(\lambda \right)c_{i}\left(t\right)DPF\left(\lambda \right)d+G(\lambda )$$

where ‘*I_o_*’ is emitted light intensity, ‘*I*’ is measured light intensity, ‘ε’ is extinction coefficient, ‘d’ is the distance between source and detector, ‘*DPF*’ is differential path length factor, ‘*G*’ is scattering geometric factor, ‘OD’ is optical density, HbR is deoxygenated hemoglobin, and HbO is oxygenated hemoglobin.

#### 3.2.2. Data Pre-Processing

The Liu et al. [39] method has proved its efficiency in decreasing global noise in fNIRS data. Work focusing on automated window selection method (moving window) to improve classification accuracy. For individual-level classification, the method combined with a linear discriminant classifier achieved a mean F1 score of 74.8%. It does have significant drawbacks, including measurements at the group level and not the individual level. One possible restriction is that the method requires a priori knowledge of stimulus timing, which may not be accessible in all fNIRS investigations. Another drawback is that the algorithm could not be successful in eliminating certain forms of noise, including motion artifacts, which are not related to the fNIRS data of interest pertaining to this research.

Hence, this research employs Individual Component Analysis (ICA) to evaluate separate source signals from a set of recordings where these source signals are combined in unknown proportions within the fNIRS signals. Several areas of the brain are influenced by varied electrical activity during the acquisition of fNIRS data. Initial signal separation was performed by the owners of the dataset used in this research, Nagels-Coune et al. [14]. They have used SNR as the criteria to select the channels. Further signal separation with ICA is used for artifact noise removal.

These source signals must demonstrate both statistical independence and non-Gaussian properties for efficient separation using ICA and reconstruction following noise reduction. Following that, the data are represented as linear combinations of several elements coming from sources that are located in a given area. The objective is to generate N distinct source signals from N linearly mixed signals obtained through N electrodes. Accordingly, the acquired signal “x” is

(2) x=As

where the unknown mixing matrix is *A* and statistically independent sources are denoted as ‘*s*’. The reconstructed or noise removal signal ‘*y*’ is

(3) y=Wx

Estimating a spatial filter matrix *W*, which is effectively the pseudo-inverse of *A*, is the most difficult problem in ICA. Non-Gaussianity must be increased to obtain independent components W and x.  The implementation of ICA is conducted with three different ICA techniques (Fast ICA, Picard, Infomax). Out of these three methods, Picard’s method gives better signal separation.

#### 3.2.3. Feature Extraction

Finding the brain regions that are responding to a stimulus and becoming active is the usual method for connecting the fNIRS signal to a particular event or task. The time domain-based features like mean, variance, skewness, and kurtosis are associated with activities extracted. Power spectral density (PSD) is used as the frequency domain feature.

#### Time Domain Features:

Statistical metrics such as mean, variance, skewness, and kurtosis are useful features that best describe the distribution of data and summarize the essential properties of to discover trends or outliers that may be of interest.

Mean: It is an indicator of the central tendency that reveals where the data are concentrated. The mean can be used to determine whether or not the data are slanted as in Equation (4).

(4) $$\mu =\frac{1}{N}\sum_{i=1}^{N}x_{i}$$

Variance: The variance of a dataset as measured by Equation (5) measures how much the data deviates from the mean. It represents the degree of variation in the dataset. High variation indicates that the data are distributed throughout a wider range, whereas low variance indicates the reverse.

(5) $$\alpha^{2}=\frac{1}{N}\sum_{i=1}^{N}{\left(x_{i}-\mu \right)}^{2}$$

Skewness: Skewness, as demonstrated in Equation (6), measures the asymmetry of the distribution. Equation (5)’s expression for skewness quantifies the distribution’s asymmetry. A longer tail is shown on the right by a positive value and on the left by a negative value. A useful statistic for determining if a dataset is normal is skewness.

(6) $$S=\frac{\frac{1}{N}\sum_{i=1}^{N}{\left(x_{i}-µ\right)}^{3}}{{\left(\frac{1}{N-1}\sum_{i=1}^{N}\left({\left(x_{i}-µ\right)}^{2}\right)\right)}^{\frac{3}{2}}}\;$$

Kurtosis: Kurtosis as in Equation (6) is a measure of the distribution’s peakness. It calculates the degree of data concentration around the mean. High kurtosis values imply that the data are strongly concentrated around the mean, whereas low kurtosis values indicate that the data are more evenly distributed.

(7) $$K=\frac{\frac{1}{N}\sum_{i=1}^{N}{\left(x_{i}-µ\right)}^{4}}{{\left(\frac{1}{N-1}\sum_{i=1}^{N}\left({\left(x_{i}-µ\right)}^{2}\right)\right)}^{2}}-3$$

#### Frequency Domain Features:

In the frequency domain, power spectral density (PSD) is the prime metric to analyze the data distribution. It gives the estimate of the spectral density. In this research, frequency domain analysis was conducted separately for HbO and HbR data.

Maximum frequency, the frequency with the greatest magnitude of the PSD of the highest average power in the full-width-half-max (FWHM) band, which is taken as frequency domain features.

#### New Feature Generation:

The new proposed fusion-based feature generation algorithm is given below as Algorithm 1. Under the proposed approach, combined Hjorth parameters, Hilbert transform, symlet, and wavelet analysis are used for new feature generations. This combination provides activity detection, especially for non-stationary signals like fNIRS which has varying activity and frequency content. Hjorth parameters are used to find statistical descriptors for signal activity, mobility, and complexity. Wavelets are used to obtain time-localized information and detect the nature of activities at specific frequencies and times, which is essential for brain activity detection. Symlet wavelets are also used to extract edge and texture features. Hilbert transform is used for capturing the instantaneous frequency or phase information of a signal.

Data from 14 channels were used to generate the fused feature set, leaving out 4 out of the 18 channels.
**Algorithm 1**: Wavelet, Hilbert, and Hjorth parameters-based new feature generation (WHHPBNFG) Step 1: Get 14 channel data f[i] for i = 1, 2, … 14.Step 2: Find Hjorth parameter activity for 14 channel data $\alpha \left[i\right]=var\left[f\left[i\right]\right)=\sum_{i=1}^{N}{\left(f\left[i\right]-\bar{f\left[i\right]}\right)}^{2}/(N-1)$ for i = 1, 2, … 14which indicates the power level of the signal.Step 3: Find Hjorth parameter mobility $\beta \left[i\right]=\sqrt{var(df[i])/dt)/\alpha \left[i\right]}$ for i = 1, 2, … 14which indicates the mean frequency or dominant oscillation rate.Step 4: Find wavelet decomposition of the 14-channel signal at M level decomposition–Daubechies wavelet${\omega [i]}_{j}=wavelet\_decom\_m\left[f\left[i\right]\right]$ for i = 1, 2, … 14, j = 1, 2, … M.Step 5: Remove extreme 25% wavelet coefficient values from all channels.${\omega n[i]}_{j}=remove_{25}\%\%({\omega [i]}_{j})$Step 6: Find average of wavelet coefficients for every channel and make it the weight value for the channel.$w_{i}=1/K\sum_{j}^{K}{\omega n[i]}_{j}$Step 7: Find a new feature set by using Hjorth parameters and weight value.$f_{new}=w_{i}*f\left[i\right]+\alpha \left[i\right]*f\left[i\right]+\beta \left[i\right]*f\left[i\right]$Step 8: Apply symlet transform on input signal $f\left[i\right]$$s\left[i\right]=symlet(f\left[i\right])$.Step 9: Apply Hilbert transform on input signal $f\left[i\right]$$h\left[i\right]=hilbert(f\left[i\right])$.Step 10: Find second new feature using step 8 and step 9 results.$f_{new1}=w_{i}*h\left[i\right]+w_{i}*s\left[i\right]$

Hjorth parameter activity and mobility are used to capture the activity and dominate the frequency of oscillation in steps 2 and 3. Steps 4 and 5 employ wavelet transform-based noise removal and activity detection, and Step 7 calculates the new feature based on Hjorth parameters and wavelet transform weight values. Step 10 calculates another new feature set using symlet and Hilbert transform.

Here, the new feature $f_{new}\;$ is generated by weighting the signal values by the average wavelet coefficient (average is calculated after removing the 25% insignificant coefficient) and Hjorth parameters. The weight value of the average wavelet coefficient is generated by removing the extreme 25% wavelet coefficient to remove artifacts in the frequency domain. Those artifacts removed coefficients at M-level decomposition as the indicator of the motor activity. When we weigh the signal by average wavelet coefficients channel-wise, that channel and data points that have activity will be included in the new feature but those not having activity will be suppressed. This will improve the accuracy of the classification.

A similar effect is carried out in the time domain when we weigh the signal by the Hjorth parameters of activity and mobility. The activity parameter of Hjorth indicates the power level of the signal. When there is a motor activity in the fNIRS signal, the power level will be more. So, the activity parameter is a probability indicator of motor activity. The mobility parameter of Hjorth indicates the mean frequency or dominant oscillation rate which is also a probability indicator for motor activity in the fNIRS signal. If there is motor activity in the fNIRS signal, there will be a more dominant oscillation rate. When fNIRS signal channels are weighted by the Hjorth parameters of activity and mobility, only those channels and data points that are probable to indicate the motor activity will be suppressed, with only those channels having no activity being accommodated in the new feature generation.

The second set of new features is generated by

$$f_{new1}=w_{i}*h\left[i\right]+w_{i}*s\left[i\right]$$

Here, the new feature $f_{new1}\;$ is generated by weighting the Hilbert transform and symlet transform signal values by the average wavelet coefficient.

$f_{new1}$ will capture only significant edge and texture features which is extracted from symlet wavelets while weighting its average wavelet transform coefficient.

Similarly, only significant instantaneous frequency or phase information is extracted from Hilbert transformed signal, whereas its average wavelet transform coefficients are weighted.

#### 3.2.4. Classifiers for Detecting fNIRS Signal

The features extracted in the time domain, frequency domain, and new feature generation act as the input for machine learning classifiers that classify the two tasks (mental drawing and spatial navigation). The classifiers implemented in this research include LDA, KNN, LGBM, and XGBOOST. Optimizing separation between classes and minimizing variance within each class is the main goal of latent discriminant analysis (LDA). This is achieved by maintaining class separability while projecting the data onto a lower-dimensional space.

KNN is a non-parametric method, meaning it does not assume anything about the data’s underlying distribution. The fundamental idea of k-NN is to find the k data points that are closest to a given test point in the feature space, and then use their labels or values to provide a projection or estimate for the requested position. The algorithm’s performance can be enhanced by adjusting the hyperparameter k. When the decision boundary is very non-linear and the data are high-dimensional, KNN performs well.

Gradient-based one-side sampling (GOSS), a unique feature management technique, is included in the Light Gradient-Boosting Machine (LGBM) and is capable of handling categorical information. Using gradient information, it determines a selection of the most pertinent category characteristics. A number of regularization techniques, including L1 and L2 regularization, are supported by LGBM and can help to minimize overfitting and improve the generalization capabilities of the model. XGBOOST is created to improve gradient boosting’s effectiveness and prediction accuracy. It builds an ensemble of decision trees and iteratively improves their predictions. XGBOOST is computationally efficient because it uses regularization approaches to prevent overfitting, accommodates missing data, and supports parallel processing.

## 4. Results

All the algorithms were implemented in three different phases, namely data preprocessing, analysis of data for feature extraction, and two motor tasks classification.

### 4.1. fNIRS Data Visualization

**Figure 3:**
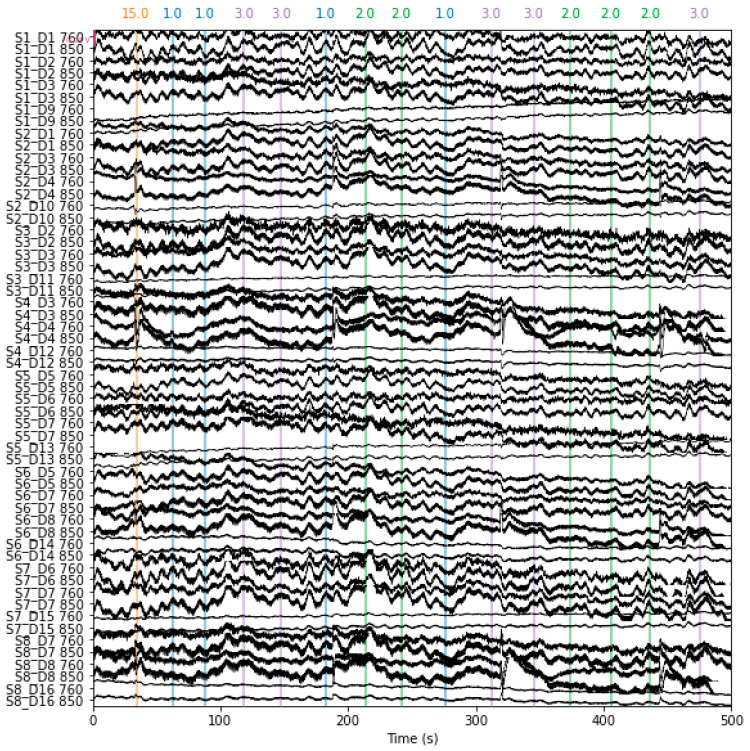
Raw fNIRS data with channel name as leftmost side in the y-axis for the duration of 500 s. The numbers on the top 15 represent the unwanted eliminated time data: 15 s data are eliminated and numbers 1, 2, and 3 indicate no activity, mental drawing, and spatial navigation activity, respectively.

**Figure 4:**
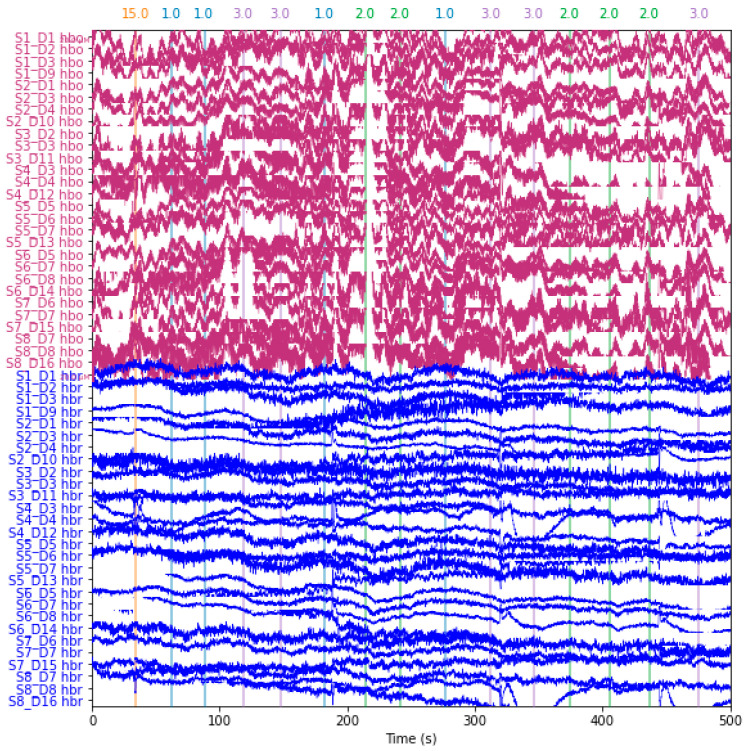
Hemoglobin-concentrated data: Applying modified Beer–Lambert law HBo and HBr components are extracted with a channel name as the leftmost side in the *y*-axis for the duration of 500 s. The numbers on the top 15 represent the unwanted eliminated time data: 15 s data are eliminated and numbers 1, 2, and 3 indicate no activity, mental drawing, and spatial navigation activity, respectively.

First, raw wavelengths were converted to optical densities in the pre-processing phase. The modified Beer–Lambert equation was then used to convert the optical density data from Figure 3 to values for oxygenated hemoglobin (HbO) and deoxygenated hemoglobin (HbR), as shown in Figure 4. ICA methods were implemented to remove noise artifacts.

### 4.2. Pre-Processed Data Visualization

**Figure 5:**
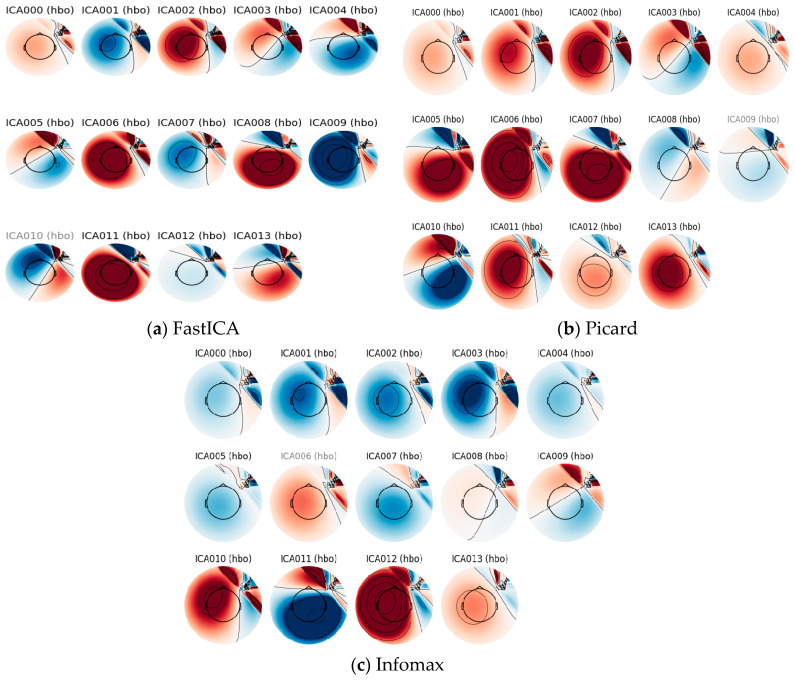
Analysis of ICA decomposition (presenting first 13 components) for different methods (Red—HbO, Blue—HbR): (**a**) FastICA, (**b**) Picard, (**c**) Infomax.

Pre-processing the data is the initial stage for identifying particular events or patterns of activity in the data. This includes reducing noise, adjusting for motion abnormalities, and normalizing the data. As discussed in the previous methodology section, three different ICA methods were implemented to remove noise artifacts. The visual representations of these three methods for the various channels are depicted in Figure 5a–c.

### 4.3. Results of Data Analysis and Feature Extraction

**Figure 6:**
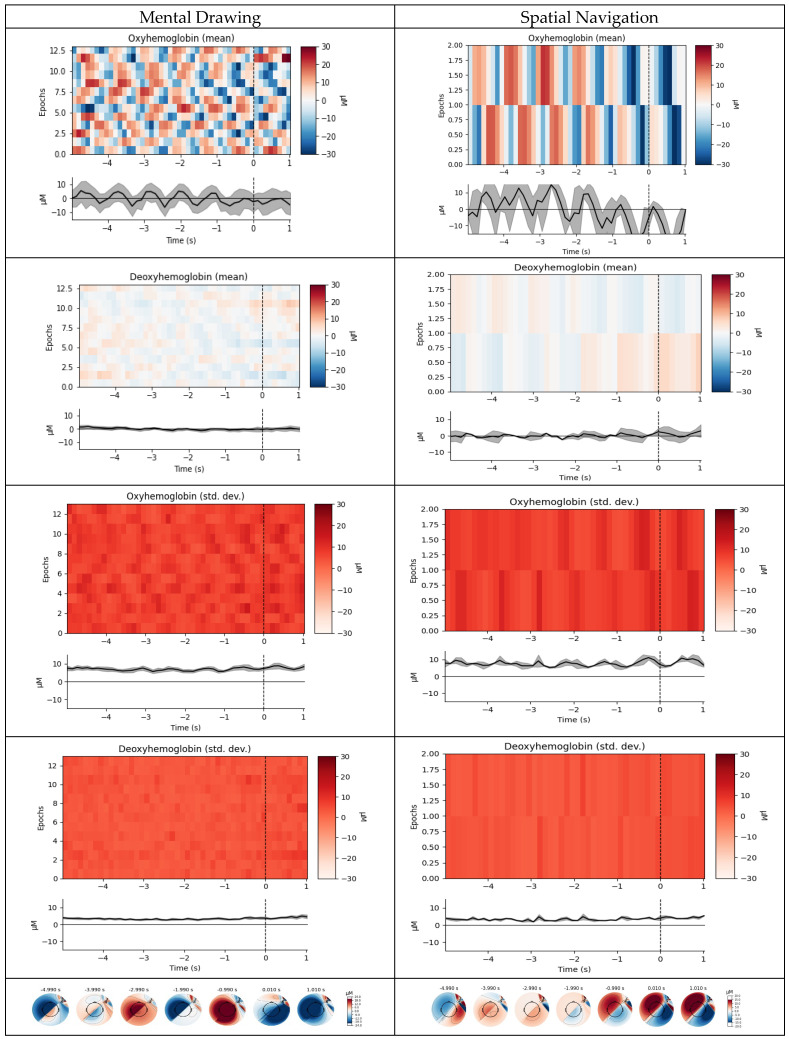
Plot of events acquired from time domain feature of mean and standard deviation: metal drawing has activity peak for every second but spatial navigation has a peak of less than one second.

In the time and frequency domains, classical feature extraction is used in addition to the new feature generated. The features of the motor activities (events) associated with spatial navigation (SN) and mental drawing (MD) were taken from pre-processed data and presented in this work. The topological maps are displayed for these occurrences, and the visualization features are calculated in terms of mean and standard deviation. The time domain data analysis is displayed in Figure 6.

**Figure 7:**
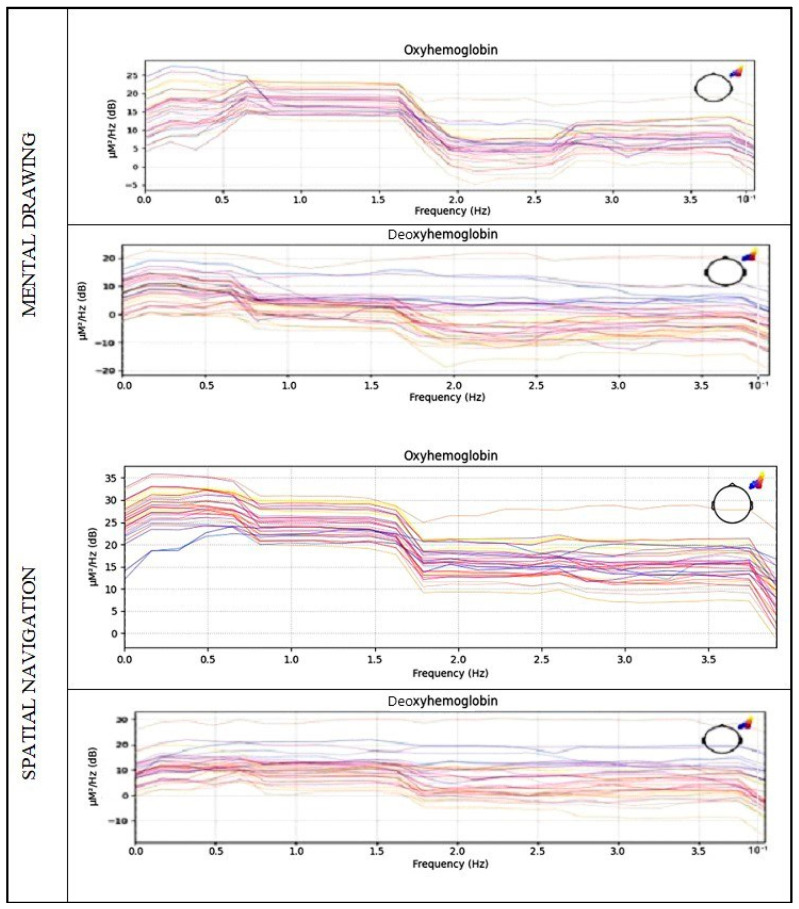
Power spectral density for mental drawing and spatial navigation; activity frequency is shown with peak value from 0.5 Hz to 1.5 Hz.

The frequency domain feature representation in terms of PSD and the corresponding maximum amplitude and frequency of the data while performing the two motor tasks is presented in Figure 7.

The first step in the new feature generation is applying wavelet transform and the signal to get the weight coefficient value. The Daubechies transform is applied to the signal and the coefficients are extracted.

**Figure 8:**
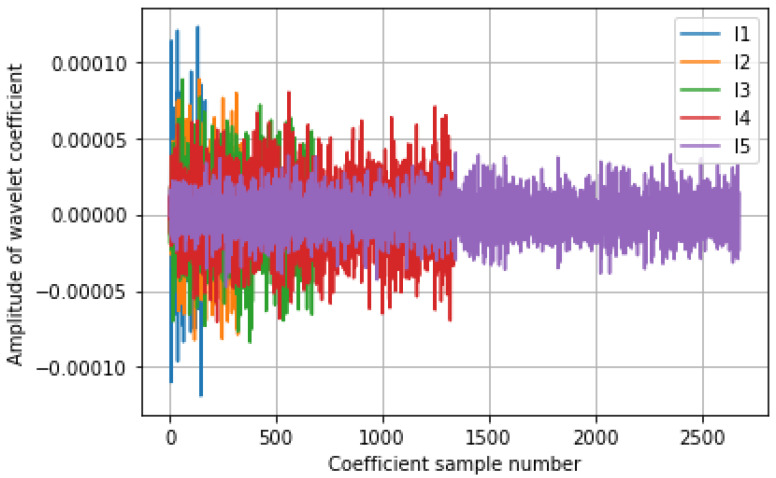
Five-level Daubechies wavelet coefficients for channel 1 data. The highest level of decomposition has a more detailed coefficient with less magnitude of noise components which can be eliminated by 25% coefficient removal.

The Daubechies wavelet is used to decompose the signal and for noise removal for new feature generation. The channel 1 coefficient is displayed in Figure 8. The signal is decomposed with five levels of decomposition using the Daubechies wavelet. Figure 8 shows at a high level of decomposition that the noise components are sitting with less amplitude, which can be removed by the 25% coefficient removal. The level 4 coefficients are used as weighting coefficients for new feature generation, which is a strong indicator of the probability of motor activity.

**Figure 9:**
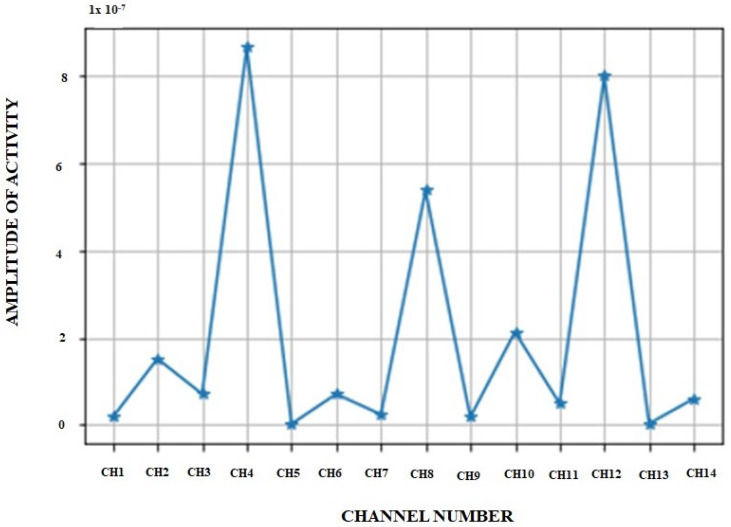
Hjorth parameter of activity for 14 channel data: three channels 4, 8, and 12 have good response.

The Hjorth parameter is used as another weighting parameter in the process of new feature generation. For interstate, its value for the activity parameter is shown in Figure 9. From Figure 9, we can observe that different channel data have different levels of activity; channel 4 and channel 11 have large activity indications. So, while generating new features, those two channels will be given more weightage.

From Figure 8 and Figure 9, it is evident that the proposed weighting (multiplicative parameters) parameter on new feature generation ensures accommodating only the data points or data channels that have the motor activity in the new future generation, and those data points and channels that do not have motor activity are suppressed or attenuated by the weighting process.

### 4.4. Results of Classification

The main objective of the work is to classify the motor activity. The classifiers implemented in this research include LDA, KNN, LGBM, and XGBOOST. The k-fold cross-validation is used for training and validation. The training was conducted using 50 epochs with a batch size of 64, taking the time domain and frequency domain features. Smartly, the new feature is also used to train the models and validate them. K-nearest neighbors (KNN) parameters used in this study are given below.

1. Number of Neighbors (k): it defines how many nearest neighbors the algorithm will consider to make a prediction; for this research work, k is considered as 10.
2. Distance Metric: Common metrics are Euclidean, Manhattan, and Minkowski. This research uses the Euclidean metric.
3. Weights: Determines the weight given to neighbors. For this research work, uniform weights were used.

**Figure 10:**
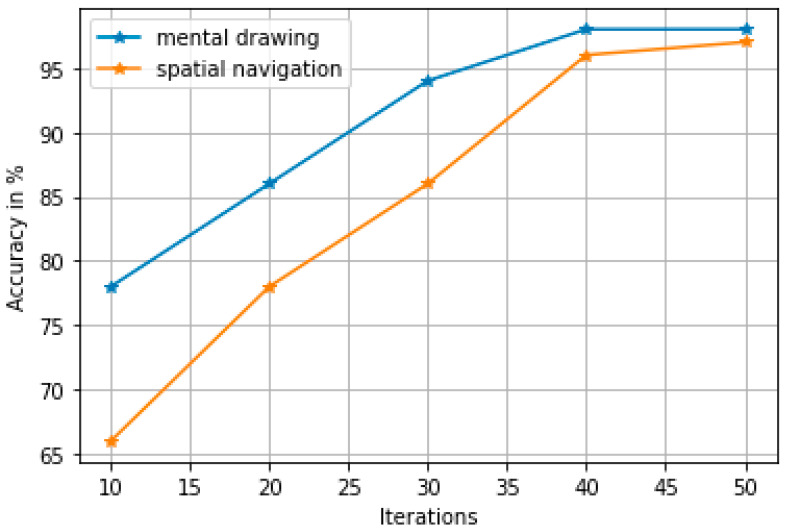
Accuracy with respect to iterations: the algorithm used in 50 iterations converges at the 40th iteration.

Figure 10 shows the accuracy of mental drawing and navigation activity for the newly generated feature set. From the figure, it is evident that the proposed new feature set can achieve 98% and 97% accuracy for mental drawing and navigation activity, respectively, at the 50th iteration.

## 5. Discussion and Comparison

From Table 1, it can be noted that the classification accuracies of the two chosen motor tasks (mental drawing and spatial navigation) are high for the new feature generation category. When classified, this gives the highest accuracy of 98% for mental drawing and 97% for spatial navigation for LGBM. When the subject is not performing the task, the brain cells are not activated and hence no oxygen flows and the signal is recorded as HbR fNIRS signal. So, accuracy levels are low for the HbR fNIRS signal.

For the classical time domain and frequency domain feature set, KNN gives the lowest accuracy for both HbO and HbR signals under both tasks (mental drawing and spatial navigation). For HbO, the accuracy levels are only 73% and 68%. Also, for HbR signals, the accuracies are less in the range of 66% and 64% for HbR signals of the two tasks. KNN appears to suffer from dimensionality loss if the number of features is too high and is more sensitive to noisy or irrelevant data. This explains why KNN has a low value. XGBOOST functions work by successively adding decision trees to the model, each of which is trained to correct errors caused by the ones that came preceding it. Using a gradient-based optimization technique, which can be modified depending on the particular task being performed, optimizes the objective function. As a result, the XGBOOST accuracy value is moderate (85% and 82% for HbO cases and 70% and 72% for HbR signals). Due to the low performance of LDA, it is assumed that the covariance matrices of all classes are equal and that the data are normally distributed. The classification accuracy is poor, with 84% and 74% for the HbO case and 72% and 70% for the HbR case, despite the dataset having fewer features overall.

As evident from the classification accuracies, LGBM performs well, as its prediction involves feature significance ranking and decision path analysis. It can be noted that LGBM gives an accuracy of 91% for mental drawing activity and 86% for spatial navigation when the task is performed (HbO signals).

Comparing the results of this research with existing research in this field, it can be noted that not many of the existing researchers have taken motor activity tasks. Most of the existing research concentrates on the resting state of the brain and very few have considered the study of motor activity. Table 2 shows the comparison of this research with existing research.

Table 2 shows accuracies of 72.3%, 91%, 70%, 85.55%, 90%, and 94.17%. It should be noted that 94.17% accuracy was implemented for “identifying biomarkers of Human Pain” which is a different dataset, whereas all others are related to motor activity like our chosen dataset. Compared with existing research works, this research shows the highest classification accuracy of 98% except for the work of Fernandez et al. [40], as their study is related to human pain biomarker identification and not motor activity. This research has implemented machine learning algorithms for classifying motor activities. Whereas existing research has taken different motor activities, this research focused on mental drawing (MD) and spatial navigation (SN).

## 6. Statistical Test Result

The Mann–Whitney U and Kruskal–Wallis H non-parametric tests were carried out for validation of the proposed method. The non-parametric version of the Student *t*-test is known as the Mann–Whitney U test, and it is used to compare independent data samples among individuals. Every test computes a test statistic, which needs to be interpreted with a certain level of familiarity with statistics as well as a more in-depth understanding of the statistical test specifically. The *p*-value, which is returned by tests, can be utilized to interpret the results of the test. The *p*-value can be thought of as the probability of observing the two data samples given the null hypothesis, which states that the two samples were drawn from a population with the same distribution. This is the base assumption.

Within the framework of a predetermined significance level with 5.0%, i.e., 0.05, the value for alpha is considered. If the *p*-value is lower than the significance level, the test indicates that there is sufficient evidence to reject the null hypothesis and that the samples were most likely drawn from populations that had different distributions.

Here, for the given problem, the null and alternate hypotheses for the Mann–Whitney U test are

**H0.**  *Sample distributions are equal, i.e., no motor activity*.

**H1.**  *Sample distributions are not equal, i.e., there is motor activity*.

The above hypotheses are tested by the Mann–Whitney U test and the results are given in Table 3.

To test whether the new feature data belong to motor activity, or mental drawing (MD) motor activity, or spatial navigation (SN) motor activity, the three-class new feature data are applied to the Kruskal–Wallis H test. The Kruskal–Wallis test is a non-parametric variant of the analysis of variance (ANOVA) test, which in its abbreviated form, is known as the one-way test. Ascertaining whether or not more than two independent samples have a different distribution can be accomplished through the utilization of this test. When compared to the Mann–Whitney U test, it can be considered the generalization of that test.

Three samples of feature data from 1. no motor activity; 2. mental drawing (MD); and 3. spatial navigation (SN) are fed to the Kruskal–Wallis test and the results are observed. Table 3 shows the Mann–Whitney U test and Kruskal–Wallis H Test results.

The statistical test results with *p*-value < 0.05 prove that the null hypothesis can be rejected, i.e., the datasets are not from the same distribution, which means the proposed new feature-generated data ensure the different motor activity data will be in different distributions from non-motor data distribution such that it can be classified accurately.

## 7. Conclusions and Future Work

This research analyzed two different motor activities (mental drawing and spatial navigation) among 18 healthy subjects using fNIRS data. The HbO and HbR signals acquired were pre-processed with three types of independent component analysis, namely FastICA, Picard, and Infomax methods. The proposed new novel feature generation improved the accuracy of classification. The new novel feature was validated with the Mann–Whitney U and Kruskal–Wallis H non-parametric test. That test proves that the new feature generated ensures different distributions of data points for no activity, mental drawing (MD) motor activity, or spatial navigation (SN) motor activity class of data which contributed more accuracy of classification while applying machine learning algorithms. Among LDA, KNN, XGBOOST, and LGBM classifiers, the highest classification accuracy was obtained for LGBM with 98% for metal drawing activity and 97% for spatial navigation activity. Since the LGBM gradient-boosting framework trains a set of weak learners, it can achieve more accuracy. Using this mental activity classifier as a base, future research will be analyzing mental drawing exercises for neurorehabilitation. Mental drawing exercises offer potential therapeutic uses for enhancing motor function in individuals with neurological diseases, which could be analyzed with fNIRS and the methodology proposed in this research.

## Figures and Tables

**Table 1:** Accuracy % of various machine learning algorithms for different tasks.

Machine Learning Algorithm	Mental Drawing—New Feature		Spatial Navigation—New Feature		Mental Drawing—Traditional Feature		Spatial Navigation—Traditional Feature	
	HbO	HbR	HbO	HbR	HbO	HbR	HbO	HbR
LDA	89	82	82	80	84	68	74	66
KNN	88	83	80	78	73	66	68	64
**LGBM**	**98**	**86**	**97**	**90**	**91**	**72**	**86**	**70**
XGBOOST	94	84	93	88	85	70	82	72

**Table 2:** Comparison of this research with existing research.

S.No	Journal Reference	Methods Employed	Results
1	Liu et al. (2021) [39]	Deep neural and CNN algorithm classification using individual based time window (ITWS).	The average F1 score attained by the ITWS algorithm was **73.2%**.
2	Fernandez Rojas et al. (2019) [40]	Three algorithms for machine learning. Support vector machines linear discriminant algorithm nearest neighbor for identifying bio markers of human pain.	The model’s SVM shows good performance, with accuracy of **94.17%**.
3	Behboodi et al. (2019) [41]	Performance of increased sensitivity and specificity using seed-based machine learning model.	Artificial network model performance yielded the best prediction, with **91%**.
4	Zheng et al. (2019) [42]	The analysis uses classifiers like shrinkage algorithms, common spatial pattern (CSP)-based techniques, and resting-state independent component analysis (RSICA).	The classification accuracy of RSICA obtained is above **70%** for all spectral datasets.
5	Eken (2021) [43]	A prediction system that utilizes the use of the Dynamic Time Warping algorithm and Pearson’s correlation was developed for calculating functional connectivity.	The results show the proposed model gives the highest accuracy of **85.55%**.
6	Hramov et al. (2020) [7]	Examination of fNIRS data collected during hypothetical and actual movements.	**90%** classification accuracy of motor imagery.
7	Proposed research work	Analysis of fNIRS data for motor activity with new feature generation.	***98% classification accuracy of mental drawing and 97%* spatial navigation**.

**Table 3:** Non-parametric statistical test result.

Test	Test Static	*p*-Value
Mann–Whitney U test	6.051	0.049
Kruskal–Wallis H test	10.076	0.036

## Data Availability

The original contributions presented in the study are included in the article, further inquiries can be directed to the corresponding author/s.
